# Investigating the differences between females perceive same-gender and heterogender sex robots regarding adoption and intentions

**DOI:** 10.3389/fpsyg.2022.922108

**Published:** 2022-08-19

**Authors:** Yuanjun Li

**Affiliations:** ASBS, University of Glasgow, Glasgow, United Kingdom

**Keywords:** artificial intelligence, psychology, human–robot interactions, marketing insights, consumer behavior

## Abstract

The market for sex robots is on the rise with the development of human–computer interaction. However, most sex robots on the market are presented as male-friendly products. This issue may limit and hinder females' adoption and utilization of sex robots. This paper was to take females as the research subjects exploring and verifying several concerns based on previous theories and to conduct primary research and quantitative method to investigate: (i) how females differently perceive same-gender and heterogender sex robots; (ii) their attitudes and the knowledge or definition of sex robots; and (iii) their intention of adopting heterogender robots. This study confirmed several previous theories and provided new findings and insights. Females are more likely to feel threatened by the presence of same-gender sex robots. Their negative attitudes are related to the way that sex robots exist. They are jealous of same-gender sex robots; nevertheless, this should not be attributed to their negative perception of sex robots since they also have positive perceptions and intentions to adopt a sex robot. They define sex robots more as sexual products than as engaging in the prostitution industry.

## Introduction

Sex robots were designed to satisfy sexuality. Scholars believe the concept of human–sexbot relationships is rising (Levy, 2009; Pearson, 2015; Langcaster-James and Bentley, 2018). Levy (2009) was the first scholar to discuss the future development of sex robots (sexbot). He believes practicing and interacting with sex robots will be a routine by 2050 (Levy, 2009).

However, Richardson (2015) spearheaded the Campaign Against Sex Robots (CASR) to oppose sex robots' development strongly. It critiques Levy's (2009) Love and Sex with Robots. Richardson (2015) expects to prove harmful properties between individuals and sex robots, similar to the relationship and properties between customers and sex workers. Danaher et al. (2018) disagree with the analogy between sex robots with sex workers. Sex workers are highly conscious humans; sex robots should be perceived as alternatives to sex products rather than sex workers (Danaher et al., 2018). The analogy can be considered a type of objectification of females.

A gap can be found by reviewing the debates that CASR's opposition to sex robots is based on the opposition to prostitution and the objectification of women. This boycott is considered to be from a female perspective. It might be based on feminism (Hancock, 2020). Besides, what emerged from the debates was that females may have different perceptions of sex robots than males. Numerous studies could prove this (Baumeister et al., 2001; Petersen and Hyde, 2010; Maas et al., 2018). Males are more optimistic about the advent of sex robots than females (Scheutz and Arnold, 2016). These differences may be related to the psychological characteristics of males and females.

On the contrary, most sex robots on the market are presented as male-friendly products (Danaher and McArthur, 2017), for example, Realdoll, a company that sells sex dolls and sex robots, specializes in creating sex robots; however, their first male sex robot, Henry, is still unavailable on the market. Richardson (2015) argues that the development of sex robots should not only be confined to female sex robots. However, the overdevelopment of female robots may limit and hinder females' adoption and utilization of sex robots. It drives difficulties in studying how females differently perceive sex robots of different genders.

Harper and Lievesley (2020) argue that there is a lack of analyses of the characteristics or behaviors of the owners. Thus, no standard measurement of the attitudes toward sex robots is available. Besides, there is a lack of clarity in previous studies on the relationship between sex robots' genders and females' perceptions. There is also a lack of investigation into females' attitudes toward sex robots.

Therefore, it is significant to investigate females' psychology and whether they might be associated with other aspects of their attitudes and behavior. For example, are females' negative attitudes toward sex robots related to the unavailability of male sex robots? Could female-friendly sex robots improve the attitudes of females adopting sex robots? Do they agree with certain advantages that sex robots could provide and have concerns about the disadvantages of sex robots? How do they define sex robots? Do they perceive sex robots as an engagement with the prostitute industry, as Richard criticized?

Therefore, the purpose of this study was to take females as the research subject to explore and verify the concerns based on previous debates and theories. This study could help scholars to better understand females' psychology and perceptions toward sex robots.

## Theories and hypotheses

### The theory of planned behavior

This study mainly adopted the theory of planned behavior (TPB) for the theoretical framework in this study. TPB was rooted in this study because it starts with the properties of an objective to determine attitudes, subjective norms, and perceived behavioral control. It could implicitly reflect individuals' motivations, values, and goals through their evaluations and beliefs (Ajzen, 1991). Exploring and explaining behavior is a complex process. Ajzen (1991) believes that behavior is a process in which individuals evaluate objectives based on their perception and form an attitude regarding acceptance, adoption, or utilization of the objectives. Attitude leads to intentions to objectives, leading to a decision on when and how to act (Ajzen, 1991) (see Appendix 01).

However, a general inquiry into attitudes cannot predict a specific behavior of a subject. Wicker (1969) argues that the concept of attitudes reflecting behavior should be abandoned. Scholars propose a remedy for this issue in which specific behaviors in different situations, occasions, and forms of actions can be gathered as an aggregation (Fishbein and Ajzen, 1974; Epstein, 1983). In other words, potential behavioral tendencies can be effectively measured by aggregating many different single samples (Ajzen, 1991). Therefore, the measurement of a certain number of samples of subjects' attitudes can be considered valid in this study.

On the contrary, LaMorte (2019) argues that TPB did not consider variables such as threat, fear, or past experiences, which are also factors in behavioral intentions. Several studies indicate the effect of fear or threats on behavioral intention (Mcdaniel and Zeithaml, 1984; Shi and Smith, 2016; Jordan et al., 2018). Therefore, the variable “threat” or “fear” was added to this study.

### Knowledge, attitude, and behavior

The knowledge, attitude, and behavior (KAB) model asserts that behavior is affected by knowledge and attitude (Bettinghaus, 1986) (see Appendix 02). In line with this, scholars believe that knowledge is the basis of establishing attitudes and behavior, and it plays an essential role in attitudes (Evans and Durant, 1995; Xu et al., 2010; Aertsens et al., 2011).

Brucks (1985) defined subjective knowledge as individuals recognizing things based on self-rated knowledge or perceptions. Scholars believe subjective knowledge is a stronger motivation for behaviors (Selnes and Gr°nhaug, 1986; Feick et al., 1992). Therefore, this study introduced the variable “subjective knowledge.” It could measure how subjects define sex robots; in other words, how do research subjects build their subjective knowledge and understanding of sex robots through perceptions?

**Figure 1:**
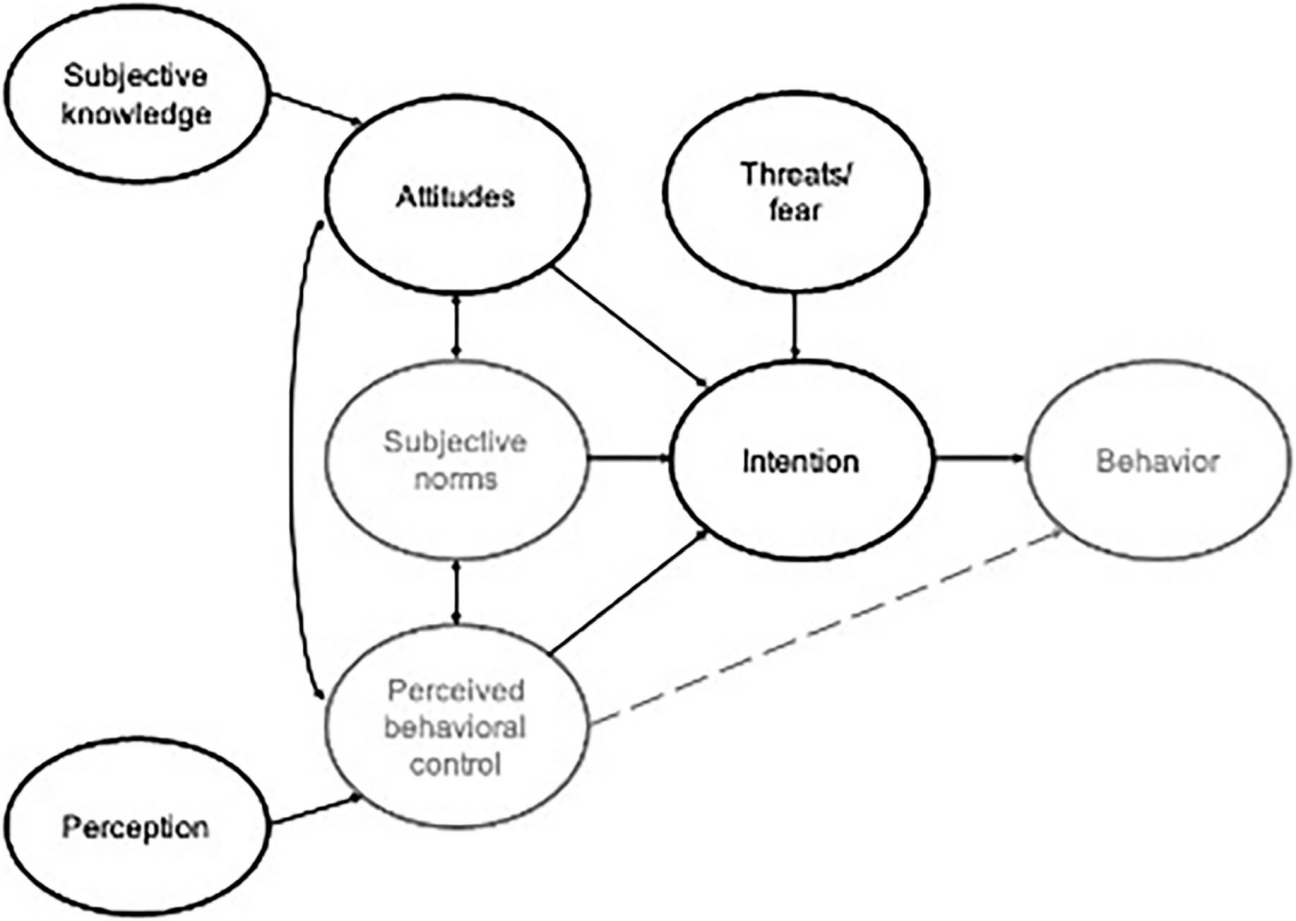
Theoretical framework. Adapt from: The theory of planned behavior (Ajzen, 1991). Knowledge, attitude, and behavior (KAB) model (Bettinghaus, 1986).

Overall, females' fear and threats from sex robots, positive and negative perceptions, attitudes or beliefs, and subjective knowledge or definition of sex robots are supposed to be found in this study, conducting that the theoretical framework combined with TPB and KAB models may provide insights into females' intentions toward sex robots in future (see Figure 1).

### Gender of sex robots

Roxxxy, the first sex robot, was designed by TrueCompanion in 2010. Although the company did not specify Roxxxy's gender, the sex robot was introduced as “she” (Cheok et al., 2016). Moreover, Realdoll, an existing company, sells sex dolls and catalogs the product's gender on its website. The sex robots Realdoll X have female physical characteristics and sexual organs (Realdoll, 2021).

Despite sex robots are not supposed to have genders, individuals assign a humanoid robot a gender depending on different macroscopic features and functions it can perform. A survey investigated by several scholars shows that the robots with longer hair and fuller lips were recognized as females, and robots with shorter hair were recognized as males (Todorov et al., 2008; Eyssel and Hegel, 2012). It is entitled sexual objectification; it has been around for a long time and occurs at a very young age of individuals (Brown et al., 2020). In other words, individuals distinguish themselves as males and females from childhood, engaging in sexually objectifying behavior.

As robots become humanoid, they also become more gendered because individuals are particularly gendered and apply gender to creating highly human-like robots (Søraa, 2017). In addition, there are also studies on the role of robot genders in human–computer interaction (Crowell et al., 2009; Siegel et al., 2009; Tay et al., 2014). Assigning gender to a robot can increase its perception of humanity (Eyssel et al., 2012; Bryant et al., 2020). Given this, gender differentiation is evocative in exploring the perception and behavior of human–robot interaction. Therefore, this study was conducted in the context of a perspective that sex robots have gender distinctions, which are same-gender (female) and heterogender (male) sex robots.

### Threats from sex robots

Previous studies have also investigated and discussed the different attitudes of men and women toward developing sex robots from a psychological or instinctive aspect (Nomura et al., 2006). Men and women have different attitudes toward robots, which might be associated with the perceptions of AI technology and human-like machines. According to McClure (2018), females are more fearful of developing artificial intelligence and robotics than men (McClure, 2018). However, this was limited to fear of human–computer interaction and did not consider factors associated with the robot's gender.

Numerous independent studies have examined male and female attitudes toward sex robots (Green et al., 2008; Siegel et al., 2009; Scheutz and Arnold, 2016). For example, Nordmo et al. (2020) conducted innovative research on how males and females perceive sex robots and platonic love robots. The results show that females have predominantly negative views toward sex robots compared to men. If the respondent's partners can obtain a sex robot, women are more jealous than men. Nordmo et al. (2020) argue that females may feel more threatened by sex robots than men. Nordmo et al. (2020) differentiated the respondents' genders and provided a meaningful insight for this study; however, the results concern different types of robots; same-gender and heterogender sex robots were not distinguished.

This study expects to distinguish males from females and male robots from female robots to verify the views, rather than different types of robots. Therefore, more substantial evidence should show how females perceive same-gender and heterogender sex robots differently. Hence, the first hypothesis was established:

*H1) Females feel more threatened by same-gender sex robots than heterogender sex robots*.

### Attitudes to the products presented way

Several studies argue that the existing sex robots are mostly female sex robots because men are the prominent supporters and consumers of sex robots while they are also the leading designers and producers of sex robots (Danaher and McArthur, 2017; Szczuka and Krämer, 2017; Oleksy and Wnuk, 2021). Richardson (2015) argues that it is the objectification of females. Nevertheless, both males and females have similar definitions of sexualized females and value them as a way to gain status (Stone et al., 2015). Females are more sensitive to this and aspire to emulate images of sexualized females because of this (McKenney and Bigler, 2014; McKenney and Band igler, 2016). Besides, Borau et al. (2021) argues that female robots are considered more acceptable than male robots because they are more humanized.

Nordmo et al. (2020) believe that females feel more threatened by sex robots than men. However, Siegel et al. (2009) argue that both men and women trusted robots of the heterogender more than the same-gender robots. It means females may perceive heterogender sex robots more positively than same-gender sex robots. Hence, a question is: Would females feel less threatened when sex robots are presented as female-friendly products?

Nevertheless, the gaps between male and female perceptions of sex robots are complex, which might be a sociological issue regarding gender status and the inequality in relationships between men and women (Danaher and McArthur, 2017; Hancock, 2020). Despite these studies being expected to explain why males and females differently perceive sex robots, the respondents' genders were not distinguished in these studies.

Oleksy and Wnuk (2021) conducted a similar survey investigating females' fear of sex robots. However, their general results did not validate the idea that females would feel less threatened if sex robots were presented as female-friendly products. After adding political factors as the moderating variables, the result was obtained among females who tend to be more liberal (Oleksy and Wnuk, 2021).

Therefore, this study was intended to continue Oleksy and Wnuk's (2021) assumption by conducting a general survey of females' perceptions of this question without moderating variables. The second hypothesis was thus formed:

*H2) Females can accept sex robots more if the sex robots are presented as female-friendly products rather than male-friendly products*.

### Advantages of sex robots (positive perceptions)

Hedonist agrees that sex robots are welcome because they can provide more hedonistic benefits (Moore, 2019). The benefits can add diversity to the sex lives and improve physical and mental health (Blanchflower and Oswald, 2004; Whipple, 2008; Brody, 2010). Scheutz and Arnold (2016) listed many benefits of sex robots (see Appendix 03). They believe the adoption and the interaction with sex robots have many advantages compared with human sexual contact, such as a low risk of disease transmission and psychological impact on the sex partner, and a sex robot is available anytime (Scheutz and Arnold, 2016, p. 254). Besides, a relatively high percentage of the subjects believe robots can improve their sex lives by adding diversity and enhancing sex experiences (Scheutz and Arnold, 2016, p. 254). In addition, females often present a hedonic purchasing motivation (Chang et al., 2004; Davis et al., 2014). It may benefit females to acquire or purchase a sex robot to obtain hedonistic benefits. Given that, a hypothesis was established as follows:

*H3) Females believe sex robots can improve their sexual experience*.

### Disadvantage (negative perceptions)

Despite there are perspectives that believe the development of sex robots has brought benefits to society, such as the benefits to the aging population and the disabled (Döring and Pöschl, 2018; Jecker, 2020). However, anti-hedonist argues that sex should be premised on procreation (Finnis, 1993) or occurs when two individuals share an emotional bond (Benatar, 2002).

Richardson (2015) argues against the development of sex robots because there are fears rooted in the concern of human relationships (Danaher and McArthur, 2017). According to Scheutz and Arnold (2016), 70% of the respondents are concerned that one of the disadvantages of sex robots is that sex robots might harm relationships between humans (e.g., abusive, controlling, and hatred for other humans). Moreover, “sex with the robot will become addictive” and “transfer unrealistic expectations to humans, leading to disappointment or abuse,” are also the main disadvantages that concern respondents (Scheutz and Arnold, 2016, p. 254).

Besides, a general view from 50% of the respondents is that they believe humans could fall in love with sex robots. About 37% of respondents argue that people will treat sex robots as human lovers (Scheutz and Arnold, 2016, p. 255). Scheutz and Arnold (2016, p. 257) argue that many of the responses thread through the concerns regarding the impact of sexual relationships. These show that the objections against the development of sex robots may be linked to relationship concerns. It may affect females' perceptions and attitudes toward sex robots. Hence, the fourth hypothesis was formed:

*H4) Females think sex robots cause relationship issues*.

### Jealousy

Szczuka and Krämer (2018) show that if females have a negative attitude toward robots in general, females react with more discomposure in response to the idea of their partner having sexual interactions with a robot. However, females' negative attitudes toward sex robots could not be the only reason for their jealousy. As discussed above, females may have negative attitudes toward sex robots; they may be concerned that sex robots influence their intimate relationships. Nevertheless, the natural females' jealousy also needs to be considered. In other words, having a negative attitude toward robots does not mean they are jealous of sex robots; females who have positive views of sex robots might also be jealous of sex robots.

Dijkstra and Buunk (1998) found that females would be intensely jealous of the scenario that their partners flirt with more beautiful women. Similarly, Yarab and Allgeier (1999) pointed out in their survey that females reported more jealousy in response to a rival with resources such as physical beauty, energy level, and fertility. Despite sex robots not having fertility resources, their appearance is considered an alternative to pornography (Danaher, 2018). They may only need to be recharged to recover the energy level. These could also cause sexual jealousy in females.

Moreover, females may have different perceptions of sex robots, and their negative attitudes might be related to the gender of existing sex robots. As argued in the previous study, females perceive discomfort with their partners being with other females rather than males (Szczuka and Krämer, 2018); the issue of sex robots might be analogous. They may particularly mind that their partner has a sex robot of the same gender as them—a female sex robot (Nordmo et al., 2020). It can be considered sexual jealousy, in which jealousy in sexual relationships is associated with suspected sexual infidelities (Duncombe et al., 2004, p. 111).

However, there are limited studies supporting females' jealousy of same-gender sex robots; thus, it is particularly significant to study the relationship between sex robot's genders and females' jealousy toward sex robots. A question was raised: are females considering their partner to have a female sex robot similar to infidelities in the relationship? It would explain the causes of jealousy beyond previous research on the relationship between negative attitudes toward robots and jealousy. It would also help researchers better understand other possibilities of females' jealousy toward sex robots. Hence, a hypothesis was formed:

*H5) Females cannot accept their partner having a female sex robot and consider it similar to infidelities in a relationship*.

### How do females define sex robots?

As mentioned above, Richardson (2015) argued that individuals possibly regard the adoption of sex robots as similar to an engagement in the prostitution industry. Nevertheless, Harper and Lievesley (2020) found that ~70% of sex robot owners define the robot as a product or a sexual companion. Only 30% of the owners utilized it for social companionship (Harper and Lievesley, 2020). It appears to be irrelevant to engaging in prostitution. It shows that individuals define a subject differently and utilize their self-rated knowledge or perceptions to recognize issues (Brucks, 1985).

Given that, there are at least two probabilities of how females define sex robots. Thus, this study expects to test the likelihood that females' knowledge or perception of sex robots is more inclined. Hence, a hypothesis was formed:

*H6) Females consider sex robots more as sex products than as similar to sex workers in the prostitution industry*.

## Materials and methods

### Methodology

Positivism was chosen as the paradigm for this study. It uses data analysis to investigate how females perceive same-gender and heterogender sex robots differently regarding their adoption and purchase intention. The literature review has found several concerns, such as females being more pessimistic about the development of sex robots; it is possible that they differently perceive sex robots of different genders. Hence, this study is more consistent with explaining the reality and the phenomenon, finding a scientific nature regarding their attitudes and intentions toward sex robots.

A deductive approach as a research logic was used for this study, which focuses on discovering measurable and observable facts and testing the differences when females perceive same-gender and heterogender sex robots regarding their perceptions, attitudes, and intentions.

Descriptive research was conducted for this study. Descriptive analysis aims to gain an accurate profile of individuals or situations (Saunders et al., 2019, p. 187), to describe a previous or existing phenomenon (Wilson, 2014). Erickson (2017) suggests that descriptive research is beneficial for gaining insights into individuals' attitudes, intentions, and behavior. It is consistent with the theoretical framework used in this study. It allows researchers to establish facts regarding a specific topic and describe the subjects' characteristics.

Quantitative research as a method of primary research is used in this study. According to Saunders et al. (2019, p. 176), researchers should consider using quantitative data associated with a deductive approach, collecting and analyzing data to test theory. This study aims to collect primary data to test the hypotheses set up from the theories; therefore, quantitative research applies.

A cross-sectional design was used for the time horizon in this study. Cross-sectional designs collect data at a single point. It is applied when researching a specific phenomenon or situation. A limitation of cross-sectional designs is that data cannot be changed and analyzed when the data are only gathered at a given time (Rafferty et al., 2015).

### Design

First, respondents' ages and genders were screened in the first section. Participants need to perceive themselves as females and must be over 18 years old. The purpose of this section was to target the group of females to ensure the data obtained are consistent with the aim of this study. Due to this study being based on females' perspectives and psychology, the group of LGBTs [an initialism that stands for lesbian, gay, bisexual, and transgender (Parent et al., 2013)] was not considered in this study.

Second, respondents were required to watch a video regarding sex robots. The content of the video includes both same-gender and heterogender sex robots. The process of producing sex robots and a few human–sexbot interaction scenarios were also involved. As mentioned above, the majority of the presence of same-gender sex robots in the market drive difficulties in studying how females differently perceive same-gender and heterogender sex robots. Therefore, the video helps to reduce the difficulties.

A Likert scale was employed in the second section of the questionnaire. The Likert (1932) scale captures how strongly a respondent feels about a given item; the distance between each candidate's value is the same (Malhotra, 2014, p. 245). The primary purpose was to measure respondents' agreement with the statements. A five-point scale was used in the questionnaire. Respondents should respond to an intensity selection from 1 (strongly disagree) to 5 (strongly agree) to specify their answers of agreement or disagreement.

Despite this, there are debates on different types of Likert scales and the meaning of “neither” selection (Jacoby and Matell, 1971; Dobson and Mothersill, 1979; Allen and Seaman, 2007; Sturgis et al., 2012). Kulas (2008) suggests that the difference was negligible, and the “neither” selection has an insignificant erosion of score appears in the result. Therefore, the five-point Likert scale is valid for this study.

Finally, nominal data were collected in the third section. Nominal data are also called nominal categories, which can be divided into several groups: for example, male or female (Rugg and Petre, 2007, p. 182). These groups do not overlap; they are unique. This section collected the demographic information of respondents' age, education level, income, and relationship status. This section aimed to identify distinctions between the respondents and provide a more specific target and segmentation for future research.

### Participants

The questionnaire was distributed on the Internet. Non-probability sampling was conducted in this study. Responses from 130 female subjects were collected as samples for the data analysis. The proportion of young respondents was as high as 72.31%. Moreover, a significant difference in proportions of single respondents (33.85%), those who were in a relationship (32.31%), and married respondents (29.99%) was not found. In addition, the education levels of the respondents were relatively high, with a large proportion of bachelor's (39.23%) and master's (46.15%) degree holders. However, their yearly income levels were under £20,000 or between £20,000 and 50,000 (see Table 1).

**Table 1:** Reliability of the survey.

**Reliability statistics**		
**Cronbach's** **alpha**	**Cronbach's alpha** **based on standardized** **items**	***N*** **of items**
0.885	0.882	22

### Measurements

There are twenty-two items, respectively, associated with the hypotheses.

#### Threats from sex robots

Threats from sex robots were measured with two items: *I feel threatened by the presence of female sex robots in society. I feel threatened by the presence of male sex robots in society*.

#### Attitudes to the products presented way

Attitudes toward sex robots were measured with nine items: *The female and male sex robots in the video gave me different feelings. I think the threat mentioned above is related to the gender of the sex robots. I think the threat mentioned above relates to the equity between men and women. I cannot accept sex robots because most sex robots are presented as male-friendly products. I accept sex robots if they are presented as female-friendly products. I would consider purchasing a male sex robot if it is available in the future. I do not feel shame about having a sex robot. I do not care how people negatively think of me if I have a male sex robot. I have certain expectations for male sex robots (such as appearance, figure, voice, etc.)*.

#### Advantage (positive perceptions)

Positive perceptions toward sex robots were measured with four items: *I believe sex robots can add diversity to my sexual experience. I believe sex robots can help to improve my sexual experience. I believe sex robots can help improve the inequality of sexual desire in relationships. I think having a male sex robot is better than having a partner*.

#### Disadvantage (negative perceptions)

Negative perceptions toward sex robots were measured with three items: *I think sex robots would negatively affect my relationship with my partner. I think sex robots would negatively affect me to have a relationship. I think sex robots would affect the human connection*.

#### Jealousy

Jealousy toward sex robots was measured with two items: *I can accept my partner to purchase a female sex robot. I consider my partner's consumption and the use of sex robots similar to cheating in a relationship*.

#### Females' knowledge (definition) of sex robots

The definition of sex robots was measured with two items: *I consider my partner's consumption, and the use of sex robots is similar to engaging in the prostitution industry. I consider sex robots more as sex products such as vibrators and sex dolls*.

### Data analysis

Descriptive analysis as the analysis method was mainly conducted in this study. First, the reliability of the survey was analyzed by applying Cronbach's alpha coefficient. The reliability of a survey refers to whether an instrument can be consistently interpreted across different situations (Field, 2015, p. 12). The items on a scale need to be internally consistent and measure the same task. Cronbach's alpha is a typical coefficient used for assessing the internal consistency of a survey (Cronbach, 1951).

Second, exploratory factor analysis was conducted to understand the structure of a set of variables. It can be used to reduce the set of variables in a dataset. It is used to measure whether the item design was reasonable and reflects the validity of the questionnaire (Field, 2015, p. 666).

Third, statistical hypothesis testing based on the theories might be able to verify the theories. Student's *t*-test as a hypothesis testing method was carried out in this study, allowing testing of an assumption applicable to a population. Student's *t*-test in this study includes one-sample *t*-tests and a paired-sample *t*-test. About 5% was used as the significance level. H1 was tested by paired-sample *t*-test because the difference can be identified from the degree to which females were threatened by same-gender and heterogender sex robots. One sample *t*-test was used for H2–H6.

One sample *t*-test allows the mean of the test variables is compared against a test value. The test value is a hypothesized value of the mean in the population (Kent State University, 2021). It may come from a literature review or a standard. “3” as the test value was used in this study because it reflects the neutral perspectives of the respondents. If a mean of a test variable is greater or less than the test value, it can be considered meaningful; in other words, the difference between the mean of each variable and the neutral test value “3” could measure the central tendency of the respondents' attitudes.

Finally, a descriptive statistic or an analysis was conducted for each hypothesis testing result. A descriptive statistic is a method that summarizes or quantitatively describes features from a collection of information. Even though an inferential statistic such as hypothesis testing was used for data analysis to reach conclusions in this study, a descriptive statistic is also presented to summarize and analyze the sample (Christopher, 2017, p. 145–187).

## Results

### Internal reliability of the survey

**Figure 2:**
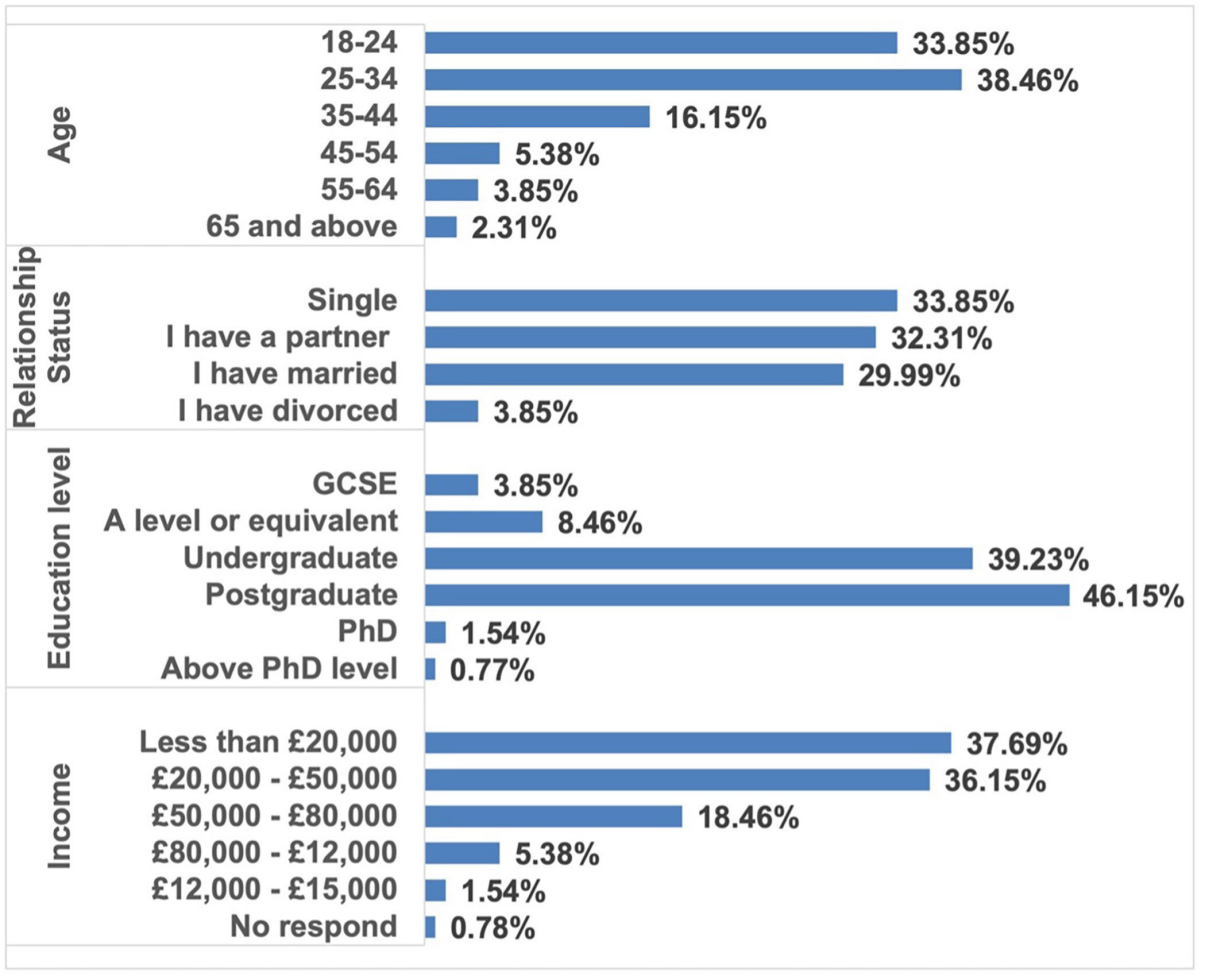
General information of respondents.

A reliability test was conducted for the items from the Likert scale in this survey. The Cronbach's alpha coefficient fell at 0.885. Cronbach's alpha was considered a coefficient between 0 and 1 (Cronbach, 1951). Nunnally and Bernstein (1994) argue that Cronbach's alpha coefficient should at least be between 0.5 and 0.69 to be adequate; more than 0.7 is considered acceptable (Nunnally and Bernstein, 1994). Therefore, the reliability of the survey was accepted (see Figure 2).

Kaiser–Meyer–Olkin (KMO) value equals 0.870, more significant than 0.5, while the significance of Bartlett's test is <0.001, which means the data were suitable for applying factor analysis (Sarstedt and Mooi, 2014) (see Table 2).

**Table 2:** KMO and Bartlett's test.

Kaiser-Meyer-Olkin measure of sampling adequacy		**0.870**
Bartlett's test of sphericity	Approx chi-square	1,694.794
	df	231
	Sig.	<0.001

Four factors were obtained with the rule of eigenvalues more significant than one. The factor score matrix presents the relationship between each factor and variable (Field, 2015, p. 673). The six hypothesis dimensions were established based on the theories that made a more detailed distinction between the factors of the results. It is consistent with the argument of Howitt and Cramer (2020) that any single study does not establish the assessment of validity. Constructing validity is a complex and ongoing process established by the pattern of results through multiple studies (Howitt and Cramer, 2020, p. 346) (see Table 3).

**Table 3:** Rotated component matrix^a^.

	**Component**			
**Items**	**F1**	**F2**	**F3**	**F4**
The female and male sex robots in the video gave me different feeling.	0.658			
I believe sex robots can add diversity to my sexual experience.	0.802			
I believe sex robots can help to improve my sexual experience.	0.864			
I believe sex robots can help to improve inequality of sexual desire in relationships.	0.720			
I think the threat mentioned above is related to the gender of the sex robots.	0.709			
I think the threat mentioned above is related to the equity between man and woman.	0.585			
I accept sex robots if they are presented as female-friendly products.	0.696			
I would consider purchasing a male sex robot if it will be available in the future.	0.752			
I do not feel shame to have a sex robot.	0.721			
I have certain expectations for male sex robots (such as appearance, figure, voice, etc.).	0.569			
I feel threatened by the presence of female sex robots in society.		0.745		
I think sex robots would negatively affect my relationship with my partner.		0.877		
I think sex robots would negatively affect me to have a relationship.		0.819		
H2.6 I think sex robots would affect human connection.		0.701		
I consider my partner's consumption and the use of sex robots as similar as cheating in a relationship.		0.669		
I cannot accept sex robot because most of the sex robots are presented as male-friendly products.		0.731		
I feel threatened by the presence of male sex robots in society.			0.652	
I do not care how people negatively think of me if I have a male sex robot.			0.673	
I think to have a male sex robot is better than having a partner.			0.673	
I can accept my partner to purchase a female sex robot.			0.754	
I consider my partner's consumption and the use of sex robots as similar as engaging in the prostitution industry.				0.670
I consider sex robots more as sex products such as vibrator, sex dolls.				0.668

Extraction method: principal component analysis.

Rotation method: varimax with Kaiser normalization.

^a^Rotation converged in six iterations.

### Hypothesis testing result

#### H1) Females feel more threatened by same-gender sex robots than heterogender sex robots

By measuring two items regarding the threats from same-gender and heterogender sex robots, paired mean difference equal to 1 (see Table 4), and a vital significance (*t* = 8.22, *p* < 0.001) between the mean of the two items. The null hypothesis was rejected; thus, H1 was accepted. Females have different perceptions of same-gender and heterogender sex robots. Their different responses to the threats from same-gender and heterogender sex robots show how was the degree to which they differently perceived the threats.

**Table 4:** Summarized hypothesis testing result.

**Paired samples** **test**	***t***	**df**	**Significance** **two-sided *p***	**Paired mean** **difference**	**95% confidence interval of** **the difference**	
					**Lower**	**Upper**
H1	8.22	129	<0.001	1.00	0.76	1.24
**One-sample test** **Test value** = **3**	***t***	**df**	**Significance** **two-sided** ***p***	**Mean** **difference**	**95% confidence interval of the** **Difference**	
					**Lower**	**Upper**
H2	8.34	129	<0.001	0.59	0.45	0.72
H3	2.93	129	0.004	0.23	0.07	0.38
H4	5.22	129	<0.001	0.49	0.30	0.67
H5	−2.42	129	0.017	−0.14	−0.26	−0.03
H6	7.57	129	<0.001	0.49	0.36	0.62

The significance level is 0.050.

Comparing the different responses shows that 59.23% of the respondents agree (35.38% strongly agree and 23.86% agree) that the presence of same-gender sex robots would threaten them, while 29.23% disagreed. On the contrary, only 19.23% of the respondents agreed that the presence of heterogender sex robots would threaten them. However, 67.69% of the respondents disagreed with it. The result indicates that females are more likely to feel threatened by the presence of same-gender sex robots (see Appendix 04).

Besides, there was a correlation between females' perception of the threats from same-gender and heterogender sex robots. It shows that the Pearson correlation coefficient (*r* = 0.32) was in the range of 0.3–0.7; thus, there was a reasonable correlation between the threats from same-gender and heterogender sex robots. Nevertheless, the correlation coefficient was closer to 0.3, which means the correlation can be considered weak (Ratner, 2009). It means females who believe same-gender sex robots threaten them may disagree with heterogender sex robots threatening them. In other words, the more sensitive they were to the threats from same-gender sex robots, the less susceptible they were to the threats from heterogender robots. However, the correlation was weak because some respondents might believe they are threatened by both same-gender and heterogender sex robots or argue that either of the two types of sex robots did not threaten them.

#### H2) Females can accept sex robots more if the sex robots are presented as female-friendly products rather than male-friendly products

There was a vital significance between the test value and the mean of the responses of H2 (*t* = 8.34, *p* < 0.001). Thus, the null hypothesis was rejected, and H2 was accepted. The answers to the questions regarding the attitudes toward how products are presented reflect females' perceptions toward the threat analyzed above and the adoption and intentions toward heterogender sex robots.

The result shows that 62.3% of the respondents agreed that the threat they perceived was related to the sex robot's gender, 66.92% of the respondents believe the threat is also associated with equality between men and women, and 63.84% of the respondents argue that they cannot accept sex robots because most sex robots are male-friendly products of the same gender as the respondents. If sex robots are presented as heterogender products, 67.69% of the respondents will accept them. These proved the theory of Siegel et al. (2009) that females have more trust in heterogender robots than in same-gender robots.

Although females have fears of sex robots and have predominantly negative perspectives on the development of sex robots (Nordmo et al., 2020), this paper found that negative attitudes might be related to how sex robots exist.

#### H3) Females believe sex robots can improve their sexual experience

There was a vital significance between the test value and the mean of the responses of H3 (*t* = 2.93, *p* = 0.004 < 0.05). The null hypothesis was rejected; thus, H3 was accepted. More than 60% of the respondents separately agreed on the advantage of sex robots with each item. The result has proved the theory that sex robots could add diversity and improve sex lives but also showed a more significant result that 71.54 and 61.54% of the female respondents in this survey believe that sex robots could provide advantages. In addition, 63.85% of the female respondents believe sex robots could address the inequality of sexual desire. Nevertheless, only 16.92% of the respondents agreed that having a heterogender sex robot is better than having a partner. It means the majority prefer human relationships to human–sexbot relationships; sex robots could not be replacements for a partner.

#### H4) Females think sex robots cause relationship issues

There is a significance between the test value and the mean of the responses of H4 (*t* = 5.22, *p* < 0.001), the null hypothesis was rejected, and H4 was accepted. About 70% of the respondents provided agreement with the statement that sex robots would affect the human connection. Approximately 60% of the respondents believe sex robots cause relationship issues. This result validates the concerns of Richardson (2015). It is highly consistent with Scheutz and Arnold's (2016) findings regarding the disadvantage of sex robots and respondents' negative perceptions and attitudes toward sex robots.

In addition to the general results from the female respondents, their relationship statuses were also distinguished. Single respondents agreed that sex robots would affect them to encounter a partner, while respondents in a relationship were considered neutral. Moreover, an interesting finding was that married respondents showed more willingness to adopt sex robots than single respondents (Appendix 05). This result could explain why respondents provided a high percentage of agreements in terms of positive perceptions toward sex robots that they believe sex robots could improve their sex lives. Nevertheless, single respondents indicated more sensitivity to the impact of sex robots on relationships.

#### H5) Females cannot accept their partner having a female sex robot and consider it similar to infidelities in a relationship

There was a vital significance between the test value and the mean of the responses of H5 (*t* = −2.42, *p* = 0.017 < 0.05). The null hypothesis was rejected; thus, H5 was accepted. Because of the jealousy, even though females showed a high degree of enthusiasm and willingness to purchase sex robots in future, the result shows a high level of rejection when the female respondents were asked whether they could accept their partner owning a sex robot. About 40 and 33.85% of the respondents strongly disagreed and disagreed with it, while only 18.46% agreed. More than half of the respondents provide agreements in which partners utilizing or purchasing sex robots is similar to cheating in a relationship (21.54% strongly agree and 46.92% agree). It may explain why females cannot accept their partners owning a sex robot.

#### H6) Females consider sex robots more as sex products than as similar to sex workers in the prostitution industry

There was a vital significance between the test value and the mean of the responses of H6 (*t* = 7.57, *p* < 0.001). The null hypothesis was rejected; thus, H6 was accepted. A more significant result than Harper and Harper and Lievesley's (2020) was presented in this study, in which the most substantial agreement (78.46%, more than the 70% in Harper and Lievesley's survey) was that females believe sex robots are a type of sex product. However, the respondents provide neutrality on the analogy between the properties of sex robots and the prostitution industry. It contradicts Richardson's theory that utilizing or purchasing sex robots might be similar to engaging in the prostitution industry (Richardson, 2015).

## Discussion

First, females differently perceive same-gender and heterogender sex robots. The theories of Richardson (2015), McClure (2018), and Nordmo et al. (2020) were proved. Females fear robotics (McClure, 2018); they feel threatened by sex robots (Richardson, 2015; Nordmo et al., 2020). In addition to the previous studies, this study found that females perceive more threats from same-gender sex robots than the threats from heterogender sex robots.

Second, this study found that females are more receptive to heterogender sex robots. It proved the theory of Siegel et al. (2009), in which females trust heterogender robots more than same-gender robots. Several statistical results support this viewpoint. For example, when respondents were asked whether they consider adopting a heterogender sex robot and whether they were ashamed to own a sex robot, most agreed and were willing to adopt one (59.23%). They would not feel ashamed (63.85%). Thus, the results proved that females are not intolerant of the development of sex robots; they may only oppose the existence of same-gender sex robots. This result might be a criticism of how sex robots are presented in society. The female subjects showed a generic result without any additional moderating variables that females hold negative attitudes because of how sex robots exist, and females present a better adoption of heterogender sex robots. It somewhat contradicts the result of Oleksy and Wnuk's (2021) survey. This result answered the question that females could accept sex robots more if the sex robots were presented as female-friendly products rather than male-friendly products.

Third, the results show that females have positive perceptions of sex robots. Females believe sex robots could improve their sex lives. Scholars such as Richardson (2015) and Nordmo et al. (2020) may have underestimated females' enthusiasm for adopting a sex robot. A certain number of females own a sex toy (Statista, 2017); they may also intend to own a sex robot if it is available in future.

Negatively, females are concerned about the influence of sex robots on their relationships. This study proved the theory of Scheutz and Arnold (2016). Females provide agreement to the harm from sex robots in human relationships (Scheutz and Arnold, 2016). They consider the behavior of utilizing or purchasing a sex robot as infidelities in their relationships. They showed intense jealousy on this issue. It proves that females negatively adopt same-gender sex robots, which is consistent with Nordmo et al.'s (2020) theory. In addition, it argued with the theory of Szczuka and Krämer (2018) that females not only express jealousy because they have a negative attitude toward sex robots but are also associated with the results of H2.

Finally, Richardson's (2015) analogy between sex robots and sex workers in prostitution may not be appropriate. The respondents disagreed on the relationship between adopting a sex robot and engaging in the prostitution industry. On the contrary, the respondents highly agree that sex robots are another alternative form of sexual products; sex robots cannot be a replacement for a partner. Consequently, the results were more likely to believe that a sex robot is a manifestation of sex products rather than being involved in the prostitution industry and equated with sex workers (see Table 5).

**Table 5:** Highlight the percentages of respondents who agree with the statements regarding the respondents' views on the knowledge attitudes and intentions toward sex robots.

**Statements**	**Agree**
Sex robots are sex products (such as vibrator, sex dolls).	78.46%
Certain expectations for male sex robots (such as appearance, figure, voice, etc.).	76.15%
Not ashamed to have a sex robot.	63.85%
Care about other's negative thoughts or judgments.	59.23%
Consider purchasing a male sex robot if it will be available in the future.	58.46%

## Conclusion and limitation

This study confirmed several previous theories and provided new findings and insights. Females are more likely to feel threatened by the presence of same-gender sex robots. Their negative attitudes are related to the way that sex robots exist. They are jealous of same-gender sex robots; nevertheless, this should not be attributed to their negative perception of sex robots since they also have positive perceptions and intentions to adopt a sex robot. They define sex robots more as sexual products than as engaging in the prostitution industry.

However, there should have many possibilities for how females define sex robots. The development of sex robots should also not be limited to sex products. Other possibilities related to sex robots should be further investigated. For example, the psychological topics of why individuals are interested in collecting sex robots or trying to treat a sex robot as a partner, more reasons females are jealous of sex robots, and other focuses associated with their positive or negative perceptions.

Since there are few products regarding sex robots in the market and the topic might be relatively perspective, the result was too general for the study and had several limitations. It is challenging to produce a satisfactory questionnaire (Bell, 2010, p. 140). Only one method of collecting data could not meet the ideal expectations. Other alternative data collection methods, such as interviews and focus groups, were not applied for this study to further strengthen the results. The segmentation of survey subjects was not thorough. For example, geographic information such as the respondent's living country was not distinguished in this survey. Besides, for demographic segmentation, LGBTQ+ was not considered, and the respondents were primarily young adults between 18 and 34, limiting the generalizability of the findings.

## Data availability statement

The original contributions presented in the study are included in the article/Supplementary material, further inquiries can be directed to the corresponding author/s.

## Ethics statement

The studies involving human participants were reviewed and approved by College Research Ethics Committee, University of Glasgow. Written informed consent for participation was not required for this study in accordance with the national legislation and the institutional requirements.

## Author contributions

The author confirms being the sole contributor of this work and has approved it for publication.

## Conflict of interest

The author declares that the research was conducted in the absence of any commercial or financial relationships that could be construed as a potential conflict of interest.

## Publisher's note

All claims expressed in this article are solely those of the authors and do not necessarily represent those of their affiliated organizations, or those of the publisher, the editors and the reviewers. Any product that may be evaluated in this article, or claim that may be made by its manufacturer, is not guaranteed or endorsed by the publisher.
