# Supplementary material for: Investigating the differences between females perceive same-gender and heterogender sex robots regarding adoption and intentions
Source: Front Psychol. 2022 Aug 19;13:922108. doi: 10.3389/fpsyg.2022.922108 (PMC9439658; doi:10.3389/fpsyg.2022.922108)

## Appendix

**Appendix 01: Adapt from the theory of planned behavior (Ajzen, 1991)*.***


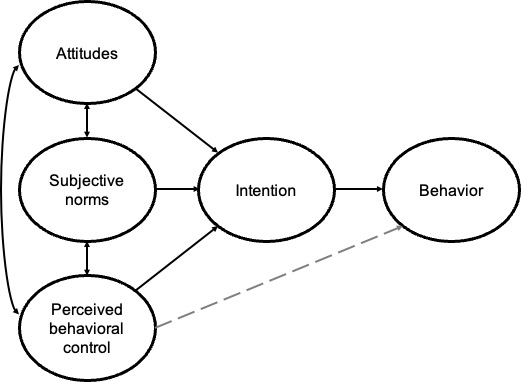


**Appendix 02: Adapt from knowledge, attitude, and behaviour (KAB) model (Bettinghaus, 1986).**


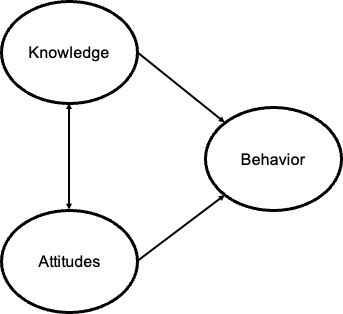


**Appendix 03: The advantage of sex robots.**

| **Advantages** | **Agree** |
| --- | --- |
| Low risk of disease transmission. | 92% |
| Available anytime. | 80% |
| Low risk of psychological impact on the sex partner. | 72% |
| Low risk of physical pain suffered from human behavior. | 62% |
| Adding diversity to sex lives and allowing people to expand sexual horizon. | 59% |
| Improve sex lives. | 54% |
| Cheaper sex. | 43% |

*Adapted from: Scheutz and Arnold (2016)*

**Appendix 04: The difference between females perceiving same-gender and heterogender sex robots.**


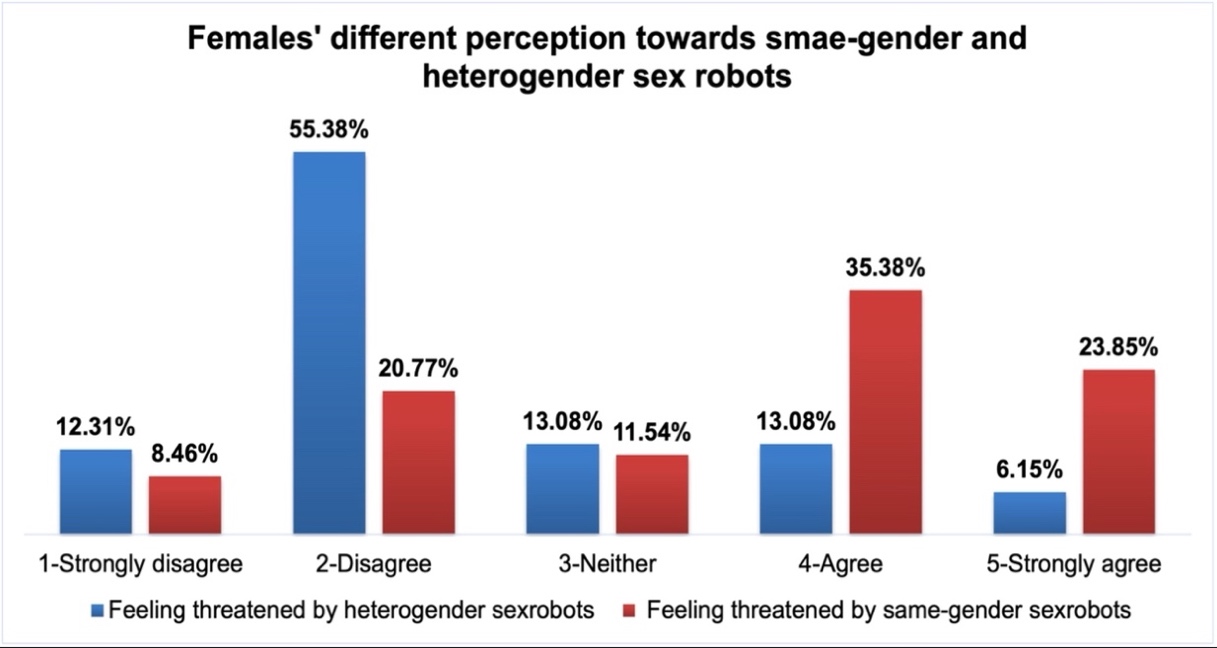


**Appendix 05: Females who agree with purchasing a male sex robot by different relationship status.**


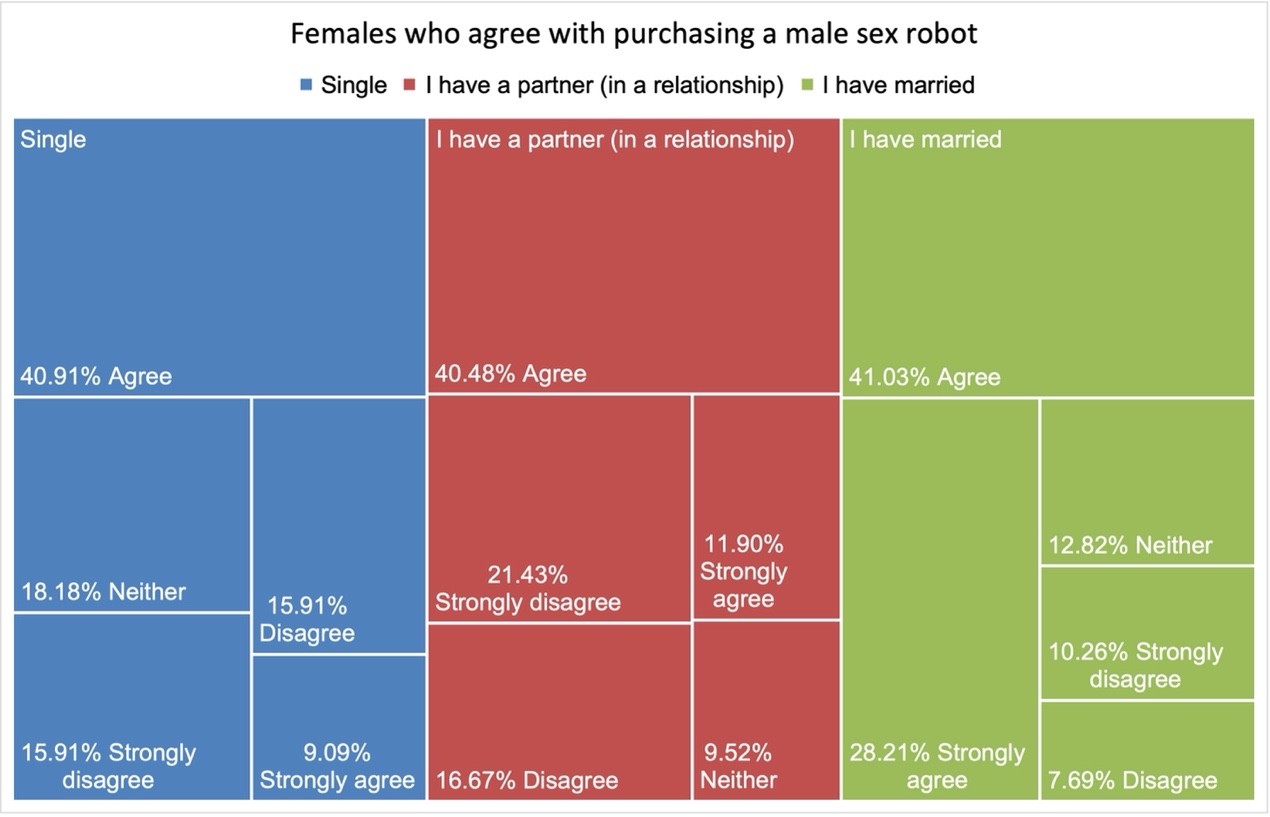

Supplement: Supplementary file 1 [file Data_Sheet_1.docx]
